# RETRACTION: Association Between Venous Leg Ulcers and Chronic Heart Failure: A Mendelian Randomization Study

**DOI:** 10.1111/iwj.70657

**Published:** 2025-04-23

## Abstract

**RETRACTION:** KeC. , ZhouQ. , LuM. , XieR. , and KongW. , “Association Between Venous Leg Ulcers and Chronic Heart Failure: A Mendelian Randomization Study,” International Wound Journal 21, no. 2 (2024): e14744, 10.1111/iwj.14744.38358070 PMC10867869

The above article, published online 15 February 2024, in Wiley Online Library (http://onlinelibrary.wiley.com/), has been retracted by agreement between the journal Editor in Chief, Professor Keith Harding; and John Wiley & Sons Ltd. Following an investigation by the publisher, all parties have concluded that this article was accepted solely on the basis of a compromised peer review process. The editors have therefore decided to retract the article. The authors did not respond to our notice regarding the retraction.

**RETRACTION:** C. Ke , Q. Zhou , M. Lu , R. Xie , and W. Kong , “Association Between Venous Leg Ulcers and Chronic Heart Failure: A Mendelian Randomization Study,” International Wound Journal 21, no. 2 (2024): e14744, 10.1111/iwj.14744.38358070 PMC10867869

The above article, published online 15 February 2024, in Wiley Online Library (http://onlinelibrary.wiley.com/), has been retracted by agreement between the journal Editor in Chief, Professor Keith Harding; and John Wiley & Sons Ltd. Following an investigation by the publisher, all parties have concluded that this article was accepted solely on the basis of a compromised peer review process. The editors have therefore decided to retract the article. The authors did not respond to our notice regarding the retraction.

